# Development of Fusion-Based Assay as a Drug Screening Platform for Nipah Virus Utilizing Baculovirus Expression Vector System

**DOI:** 10.3390/ijms25169102

**Published:** 2024-08-22

**Authors:** Indah Permata Sari, Christopher Llynard D. Ortiz, Lee-Wei Yang, Ming-Hsiang Chen, Ming-Der Perng, Tzong-Yuan Wu

**Affiliations:** 1 Institute of Molecular Medicine, College of Life Science, National Tsing Hua University, Hsinchu 30013, Taiwan; indahsk@gmail.com (I.P.S.); meanshine1982mhc@gmail.com (M.-H.C.); 2 Department of Bioscience Technology, Chung Yuan Christian University, Chung-Li, Taoyuan City 320314, Taiwan; 3 Chemical Biology and Molecular Biophysics Program, Taiwan International Graduate Program, Institute of Biological Chemistry, Academia Sinica, 128 Academia Road, Section 2, Taipei 11529, Taiwan; christopherllynardortiz@gmail.com (C.L.D.O.); lwyang@life.nthu.edu.tw (L.-W.Y.); 4 Institute of Bioinformatics and Structural Biology, National Tsing Hua University, Hsinchu 30013, Taiwan; 5 Department of Chemistry, National Tsing Hua University, Hsinchu 30013, Taiwan

**Keywords:** Nipah virus, baculovirus expression vector system, syncytium, fusion inhibitor, suramin, docking, MMGBSA

## Abstract

Nipah virus (NiV) is known to be a highly pathogenic zoonotic virus, which is included in the World Health Organization Research & Development Blueprint list of priority diseases with up to 70% mortality rate. Due to its high pathogenicity and outbreak potency, a therapeutic countermeasure against NiV is urgently needed. As NiV needs to be handled within a Biological Safety Level (BSL) 4 facility, we had developed a safe drug screening platform utilizing a baculovirus expression vector system (BEVS) based on a NiV-induced syncytium formation that could be handled within a BSL-1 facility. To reconstruct the NiV-induced syncytium formation in BEVS, two baculoviruses were generated to express recombinant proteins that are responsible for inducing the syncytium formation, including one baculovirus exhibiting co-expressed NiV fusion protein (NiV-F) and NiV attachment glycoprotein (NiV-G) and another exhibiting human EphrinB2 protein. Interestingly, syncytium formation was observed in infected insect cells when the medium was modified to have a lower pH level and supplemented with cholesterol. Fusion inhibitory properties of several compounds, such as phytochemicals and a polysulfonated naphthylamine compound, were evaluated using this platform. Among these compounds, suramin showed the highest fusion inhibitory activity against NiV-induced syncytium in the baculovirus expression system. Moreover, our in silico results provide a molecular-level glimpse of suramin’s interaction with NiV-G’s central hole and EphrinB2’s G-H loop, which could be the possible reason for its fusion inhibitory activity.

## 1. Introduction

Nipah virus (NiV) is known as a deadly zoonotic virus that belongs to the family of *Paramyxoviridae* and the genus *Henipavirus*. The viral entry is pH-independent and initiated by the attachment of viral glycoprotein (NiV-G) to the cellular receptor EphrinB2 [1], which triggers a conformational change in the fusion protein (NiV-F) and subsequently mediates the fusion between the viral and cell membrane [2] (Figure S1). This mechanism also triggers the cell-cell fusion once NiV-F and NiV-G proteins are expressed on the cell surface, which results in syncytium formation, one of NiV’s hallmarks [3].

NiV has been demonstrated to lead to a high mortality rate and is capable of infecting a wide range of hosts [4]. It was first isolated and reported to be the causative agent of viral encephalitis outbreaks in Malaysia and Singapore in 1998–1999 [5]. A total of 265 reported cases and 105 fatalities were recorded in Malaysia. Meanwhile, 11 cases with 1 fatality, involving abattoir workers who handled infected pigs imported from Malaysia, were reported in Singapore [5,6]. Since then, NiV outbreaks have been reported in India, the Philippines, and almost annually in Bangladesh [7,8,9]. Individuals infected by NiV suffer from respiratory disorders and encephalitis [10]. To date, there are approximately 650 individuals that have been affected by NiV infection, with up to a 70% mortality rate [9]. It is also worth noting that the natural reservoir of NiV, including the island flying foxes (*Pteropus hypomelanus*) and Malayan flying foxes (*Pteropus vampyrus*), is widely distributed [11,12]. Importantly, surveillance of NiV in the bat population has confirmed the presence of positive antibodies against NiV in several countries [13,14,15,16,17,18,19]. All findings on the pathogenicity and outbreak potency of NiV suggest that it is a significant threat to global health.

To date, there is no approved therapeutic or prophylactic countermeasure against NiV infection. Limited studies have demonstrated the efficacy of several nucleoside analogs against NiV infection using various animal models, including balapiravir (R1479), favipiravir (T-705), and remdesivir (GS-5734) [20,21,22]. However, nucleoside analogs have been associated with a handful of side effects, such as hepatotoxicity, vomiting, nausea, as well as respiratory toxicity [23,24]. Therefore, an alternative therapeutic countermeasure against NiV, such as an inhibitor of viral entry, is needed.

As NiV has to be handled within a Biological Safety Level (BSL) 4 facility [25], it was impractical to develop the drug screening platform using the wild-type virus. Thus, it was less labor-intensive to express NiV’s proteins using the baculovirus expression vector system (BEVS), which offers several advantages, including a large insert capacity, and the simultaneous high expression of multiple proteins [26]. BEVS-expressed proteins undergo post-translational modifications, which allow the correct protein folding, resulting in functional recombinant proteins [26]. In this study, we developed a drug screening platform based on syncytium formation using the BEVS and evaluated the antiviral activities of several compounds, specifically their fusion inhibitory properties. As the NiV-induced syncytium formation follows a similar mechanism as the viral entry, compounds with fusion inhibitory activity screened by this platform might also prevent viral entry. By utilizing this platform, we were able to screen several compounds and assess their fusion inhibitory activities against NiV. In addition, we included molecular docking and Molecular Dynamics/Molecular Mechanics with Generalized Born and Surface Area Solvation (MD/MMGBSA) results to evaluate the interaction sites of the tested compounds on NiV-G and EphrinB2.

## 2. Results and Discussion

### 2.1. NiV Membrane Proteins and EphrinB2 Were Expressed on the Surface of Infected Sf21 Cells

**Figure 1:**
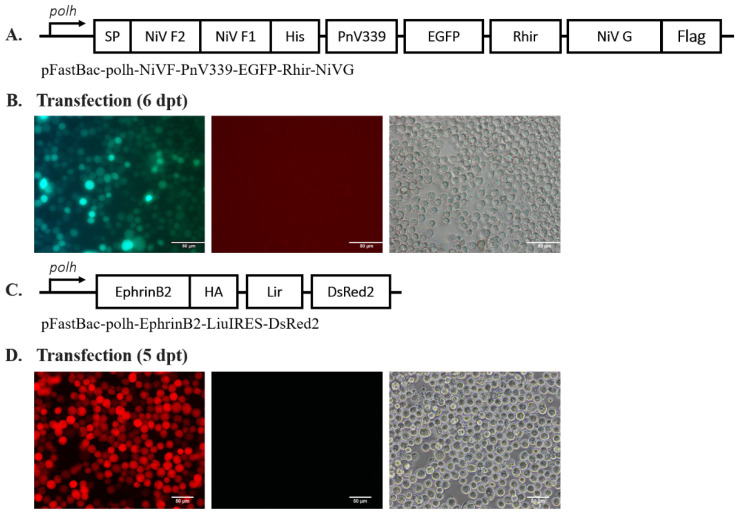
Generation of recombinant baculovirus Ac-F-EGFP-G and Ac-EphB2. (**A**) Schematic map of pFastBac-polh-NiVF-PnV339-EGFP-Rhir-NiVG. (**B**) Sf21 cells transfected with Ac-polh-NiVF-PnV339-EGFP-Rhir-NiVG bacmid were observed using the fluorescein isothiocyanate (FITC) filter at 6 dpt. Scale bar = 80 µm. (**C**) Schematic map of pFastBac-polh-EphrinB2-Lir-DsRed2. (**D**) Sf21 cells transfected with Ac-polh-EphrinB2-Lir-DsRed2 bacmid were observed using a rhodamine filter at 5 dpt. Scale bar = 50 µm.

**Figure 2:**
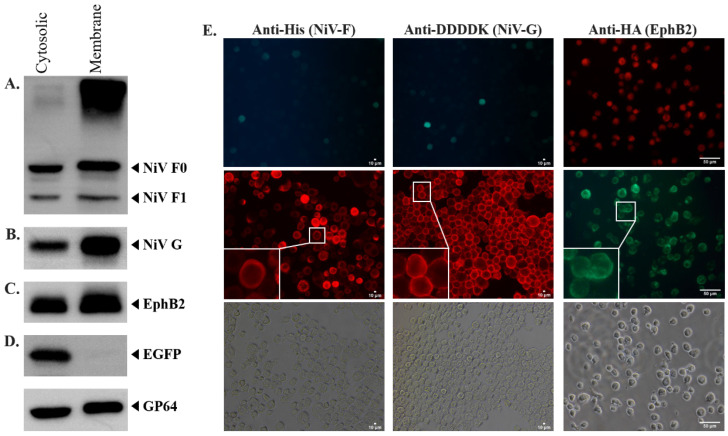
Determination of NiV-F, NiV-G, and EphrinB2 surface expression on the infected Sf21 cell membrane. (**A**) Western blot result of the cytosolic and membrane protein sample collected from Ac-F-EGFP-G-infected Sf21 cells against anti-His antibody (for detecting NiV-F), and (**B**) anti-DDDDK antibody (for detecting NiV-G). (**C**) Western blot result of the cytosolic and membrane protein sample collected from Ac-EphB2-infected Sf21 cells against anti-HA antibody (for detecting EphrinB2). (**D**) Western blot result of the cytosolic and membrane protein sample collected from Ac-EGFP-infected Sf21 cells against anti-GFP antibody. GP64 was used as an internal control and was run as a separate gel. The SDS-PAGE was carried out using 10% acrylamide gel for NiV-F and NiV-G, and 12% acrylamide gel for EphrinB2 and EGFP. (**E**) IFA results for anti-His, anti-DDDDK, and anti-HA antibodies. Scale bar = 10 µm (anti-His, anti-DDDDK), 50 µm (anti-HA).

The recombinant baculoviruses were generated with polycistronic vectors, which utilize the combination of the polyhedrin promoter (polh) with various internal ribosomal entry sites (IRES) derived from the *Perina nuda* virus (PnV339 IRES) and *Rhopalosiphum padi* virus (RhIR), as well as our chimeric IRES (Lir). The generation of recombinant baculovirus expressing NiV membrane proteins and EphrinB2 was confirmed by the positive EGFP and DsRed2 signal observed 6 and 5 days post-transfection (dpt), respectively (Figure 1B,D). Western blot analysis against anti-His and anti-DDDDK demonstrated a positive band, which suggests the successful expression of His-tagged-NiV-F and Flag(DYKDDDDK)-tagged-NiV-G (Figure 2A,B), respectively. The positive bands were higher than the expected size of NiV-F, 58 kDa [27], and NiV-G, 67 kDa [27], due to the presence of N-linked glycosylations. NiV-F and NiV-G each possess five N-linked glycosylation sites [28,29]. The removal of N-linked glycosylation from the expressed recombinant NiV-F and NiV-G by PNGase-F treatment (New England Biolabs, Ipswich, MA, USA) resulted in a positive band around the expected size of NiV-F and NiV-G (Figure S2). The expression of recombinant EphrinB2 protein was also confirmed by Western blot which showed a major band around the expected size of EphrinB2, 38 kDa (Figure 2C).

As the main purpose of the expression of NiV membrane protein, NiV-F, and NiV-G, as well as EphrinB2 was to induce syncytium formation, it was necessary to confirm the expression of the target proteins on the surface of the infected Sf21 cells. The successful surface expression of these proteins on the infected Sf21 cell membrane was necessary to allow the initial interaction between NiV-G and EphrinB2, which could subsequently trigger conformational changes in NiV-F, resulting in syncytium formation. The Western blot results demonstrate that all target proteins can be expressed in both the membrane and cytosol of the infected Sf21 cells, with a much higher proportion of them being sorted into the membrane (Figure 2A–C, lane 2). Here, the EGFP was used as the cytosolic protein control to avoid false-positive results due to contamination of the membrane protein sample with cytosolic protein. The EGFP should not be transported into the membrane of the infected Sf21 cells, thus should not be detected in the membrane. Indeed, the Western blot result shows that EGFP could only be detected in the cytosolic protein sample and was absent from the membrane (Figure 2D), suggesting that the method used to extract the membrane protein sample was sufficient to prevent the cytosolic protein from contaminating the membrane protein sample.

To further solidify the results of membrane expression of the recombinant NiV-F, NiV-G, and EphrinB2, the IFA result also suggests the successful surface expression of target recombinant proteins. The fluorescence signal shows that recombinant NiV-F, NiV-G, and EphrinB2 could be detected in fixed impermeable infected Sf21 cells using anti-His, anti-DDDDK, and anti-HA antibodies, respectively (Figure 2E), which suggests that all target recombinant proteins were successfully expressed on the membrane of the infected Sf21 cells.

### 2.2. NiV-Induced Syncytium Formation Was Observed under Low pH- and Cholesterol-Supplemented Culture Condition

**Figure 3:**
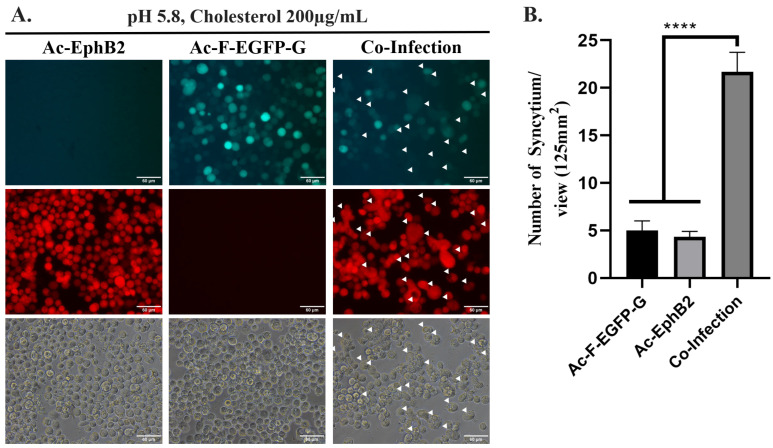
(**A**) Observation of NiV-induced syncytium in Ac-F-EGFP-G- and/or Ac-EphB2-infected Sf21 cells cultured in TNM-FH medium pH 5.8 and 200 µg/mL of cholesterol-supplemented TNM-FH at 27 °C for 4 dpi. Scale bar = 60 µm. White arrows indicate syncytium formation. (**B**) Quantification of syncytium formation in Ac-F-EGFP-G- and/or Ac-EphB2-infected Sf21 cells. Syncytium formations were quantified using three different views of the same well. Statistics were performed using one-way ANOVA, **** *p*-value of <0.0001. The data were presented as means with standard deviation.

Syncytium formation was induced by co-infecting Sf21 cells with Ac-F-EGFP-G and Ac-EphB2 under a modified culture condition (Figure 3A, indicated by white arrows). Statistically less syncytium formation was observed in individually infected Sf21 cells using either Ac-F-EGFP-G or Ac-EphB2 compared to co-infected Sf21 cells (Figure 3B). These findings suggest that the co-infection of Sf21 cells with both Ac-F-EGFP-G and Ac-EphB2 is essential for NiV-induced syncytium formation. This observation aligns with those of previous studies, which demonstrates that the expression of all three recombinant target proteins, including NiV-F, NiV-G, and EphrinB2, is required for efficient syncytium formation in insect cells [3].

To further validate this finding, we performed co-culture experiments with Sf21 cells individually infected with Ac-F-EGFP-G and Ac-EphB2 2 days post-infection (dpi). The culture medium was replaced with TNM-FH, adjusted to pH 5.8, and supplemented with 200 µg/mL of cholesterol. However, this approach did not yield conclusive results. The co-cultured, infected Sf21 cells aggregated and were unable to reattach to the wells, rendering the observation of syncytium formation unfeasible.

To facilitate the NiV-induced syncytium formation using our platform, adjustment of the culture medium’s pH and supplementation of 200 µg/mL of cholesterol at 2 dpi was necessary. The observation of NiV-induced syncytium formation occurred two days after the culture medium was replaced with fresh medium with a pH of 5.8 and supplemented with 200 µg/mL of cholesterol (Figure 3A, indicated by white arrows). In this study, all multinucleated cells were considered to be 1 syncytium (Figure S3). The combination of lower pH value and cholesterol supplementation was critical in facilitating NiV-induced syncytium formation in infected insect cells. Specifically, syncytium formation was absent when the culture medium was adjusted solely to pH 5.8 without cholesterol supplementation, or when cholesterol was supplemented at 200 µg/mL without concurrent pH reduction (Figure S4).

Cholesterol has been indicated to facilitate membrane fusion, including virus-induced membrane fusion during viral entry [30]. It increases cell membrane fluidity and flexibility, thereby promoting curvature formation [30], which is crucial during fusion pore formation. Importantly, a previous study has demonstrated that the presence of cholesterol was critical for NiV-F-induced cell-cell fusion, specifically during the fusion pore formation [31]. As insect cells cannot synthesize sterol naturally, they contain significantly less cholesterol in their membrane compared to the mammalian cell membrane [32]. Therefore, cholesterol supplementation in the culture medium of the insect cells could increase cholesterol content within the insect cell membrane [33] and trigger NiV-induced syncytium formation in infected Sf21 cells during this experiment. Nonetheless, the supplementation of cholesterol alone proved insufficient to facilitate NiV-induced syncytium formation in infected insect cells, as no syncytium formation was observed under this condition (Figure S4B).

In this study, it was apparent that a lower pH of the culture medium along with the supplementation of cholesterol was required for NiV-induced syncytium formation (Figure 3). The lower pH of the culture medium is known to trigger syncytium formation facilitated by the baculovirus envelope glycoprotein, GP64 [34]. To verify that the observed syncytia were induced by NiV membrane proteins rather than GP64, Sf21 cells were infected with a recombinant baculovirus expressing green fluorescent protein under our specific conditions. No syncytium formation was observed (Figure S5), which was expected given that the baculovirus’ GP64 requires a pH of 5.5 or lower, unlike our experimental set-up’s pH of 5.8, to successfully induce membrane fusion in insect cells [34].

The enhancement of syncytium formation at low pH has been previously observed in the BEVS expressing the Chikungunya virus membrane protein, consistent with the pH-dependent nature of Chikungunya virus membrane fusion during cell entry [35,36]. However, since NiV cell entry and NiV-induced cell-cell fusion are generally considered pH-independent processes [37], the mechanism underlying the enhancement of NiV-induced syncytium formation in BEVS at reduced pH remains unclear.

Interestingly, while NiV fusion is pH-independent, studies on Newcastle Disease Virus (NDV), another member of the Paramyxoviridae family, have shown that it can utilize different pathways for cellular entry depending on the cell line [38]. Additionally, brief exposure to low pH has been reported to increase NDV-induced cell fusion in certain cell lines, potentially by enhancing the exposure of specific protein sites critical for promoting fusion [39]. These findings raise the possibility that NiV might also utilize an alternative pathway for cell entry and syncytium formation in insect cells, potentially requiring low pH conditions for efficient fusion. Further research is needed to clarify this potential mechanism.

The proteolytic post-transcriptional modification of the NiV fusion protein was a fundamental prerequisite for NiV cell entry and NiV-induced syncytium formation [40]. The fusion protein is initially synthesized as a precursor, NiV-F0, which is transported to the cell surface in its immature form [41]. Then, the NiV-F0 protein is internalized into the cell through clathrin-mediated recycling endosomes. Within the endosomal compartment, the precursor protein undergoes cleavage by pH-dependent proteases, specifically cathepsin L and cathepsin B [37,41], resulting in the formation of mature NiV-F1 and NiV-F2 linked by a disulfide bond [40]. The mature NiV-F1-F2 complex is then transported back to the cell surface, where it can be incorporated into budding viral particles, facilitating subsequent rounds of viral infection and syncytium formation [42].

The Western blot result against the anti-His antibody shows the presence of a positive band between 40 kDa and 55 kDa (Figure 2A), which agreed with the expected size of NiV-F1 (48 kDa) [27]. The result demonstrates that the recombinant NiV-F protein could be successfully processed to form its mature conformation in infected insect cells cultured in a normal pH medium, as the homolog of cathepsinL has been reported in both AcMNPV and Sf21 cells [43,44].

### 2.3. The Compounds That Showed Fusion Inhibition against NiV

As the NiV-induced syncytium formation in Sf21 cells has been established, a total of four compounds which were previously reported to inhibit a wide range of viral infections and had demonstrated fusion inhibitory properties including Oleanolic Acid (OA), baicalein, baicalin, and suramin were screened to determine their efficacy against NiV infection. OA (3β-hydroxyolean-12-en-28-oic), a phytochemical categorized as pentacyclic triterpenoid, and its derivative have been previously demonstrated to work against influenza A virus by interfering with the fusion protein, preventing viral entry [45]. It has also been reported that OA derivatives can interact with the HR2 domain of fusion proteins of influenza A, interfering with the activity of the fusion protein during membrane fusion [45]. Baicalein (5,6,7-trihydroxyflavone) was a major flavonoid compound extracted from *Scutellaria baicalensis* [46] and was metabolized prevalently to form baicalin (5,6-dihydroxy-7-O-glucuronideflavone) [47]. Baicalin has demonstrated fusion inhibitory activity against Human Immunodeficiency Virus (HIV) type 1, most likely by preventing the interaction between the Env protein with its co-receptor [48]. Another study also indicates that baicalin exhibits antiviral properties against a Paramyxoviridae member, the Sendai virus, through the inhibition of hemagglutinin-neuraminidase activity [49], which plays a significant role during viral entry. Moreover, there is evidence suggesting that baicalein might prevent the attachment of both the Dengue virus and Japanese encephalitis virus (JEV) to host cells [50,51]. Suramin is a polysulfonated naphthylamine compound mainly used for the treatment of *Trypanosoma brucei rhodesiense* and *Onchocerca volvulus* [52].

**Figure 4:**
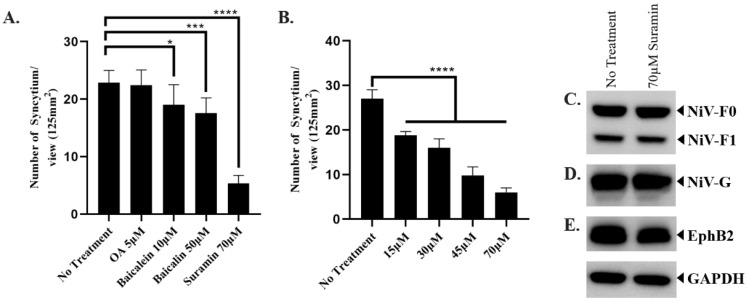
Syncytium inhibitor assay. (**A**) Screening of fusion inhibitor activity of OA, baicalein, baicalin, and suramin. (**B**) Fusion inhibitory effect of different concentrations of suramin. The statistics were performed using one-way ANOVA, * *p*-value of <0.05, *** *p*-value of <0.001, **** *p*-value of <0.0001. The data were presented as means with standard deviation. (**C**) Western blot result of protein sample collected from Ac-F-EGFP-G- and Ac-EphB2-co-infected Sf21 cells against anti-His antibody (for detecting NiV-F), (**D**) anti-DDDDK antibody (for detecting NiV-G), and (**E**) anti-HA antibody (for detecting EphrinB2). GAPDH was used as the internal control and was run separately.

Using our fusion inhibitor assay, we determined the fusion inhibitory activity of 5 µM of OA, 10 µM of baicalein, 50 µM of baicalin, and 70 µM of suramin. The concentration of each compound utilized in this study was determined based on cytotoxicity assays, where the highest concentration that did not produce a statistically significant reduction in cell viability was selected (Figure S6). Firstly, treatment with 5 µM of OA showed no statistically significant difference in NiV-induced syncytium formation compared to the untreated group tested using our platform (Figure 4A). Secondly, the treatment with 10 µM baicalein and 50 µM baicalin decreased the syncytium formation in Sf21 cells by approximately 17% and 23% with statistical significance, respectively (Figure 4A). Lastly, a statistically significant decrease in the number of syncytium/view was detected in the 70 µM suramin-treated cells (5.33 ± 1.41 syncytium/view) compared to non-treated cells (22.89 ± 2.09 syncytium/view), corresponding to an approximately 76.70 ± 7.12% decrease in NiV-induced syncytium formation under such conditions (Figure 4A). These findings show that OA failed to inhibit NiV-induced syncytium formation in infected insect cells, while the flavonoids baicalein and baicalin showed minimal fusion inhibitor activity against NiV. In contrast, suramin demonstrated statistically significant fusion inhibitor activity against NiV.

After determining that suramin is a potential candidate against NiV, different concentrations of suramin were tested to determine whether the fusion inhibitory property of suramin was dose-dependent. Treatment with 15, 30, 45, or 70 µM of suramin demonstrated that the fusion inhibitory activity of suramin was dose-dependent (Figure 4B). To determine its effect on the recombinant protein expression level, we cultured Ac-F-EGFP-G- and Ac-EphB2-co-infected Sf21 cells in the presence and absence of suramin. The result shows that there was no difference in the intensity of the band (Figure 4C–E), suggesting that suramin treatment did not cause any downregulation of NiV-F, NiV-G, or EphrinB2 expression in infected Sf21 cells, which could lead to lower syncytium formation.

Suramin exhibits robust antiviral efficacy against a diverse spectrum of viruses [52]. This broad-spectrum antiviral activity primarily stems from its capability to interfere with the viral attachment or entry into host cells, which is most likely accomplished by inhibiting the interaction between viral membrane proteins and their respective cellular receptors. Notably, suramin has been reported to hinder the attachment and entry processes of various viruses, including HIV, Herpes Simplex Virus (HSV) type I, Hepatitis C virus, Dengue virus, Severe Acute Respiratory Syndrome Coronavirus 2 (SARS-CoV-2), Enterovirus 71, Ebola virus, Chikungunya virus, and Zika virus [53,54,55,56,57,58,59]. Moreover, suramin has been reported to inhibit the replication of Dengue and Zika viruses by interacting with the viral helicase protein [60,61]. Recently, the interaction between suramin and the nucleocapsid of SARS-CoV-2 was revealed, suggesting a potential inhibitory effect on the genome packaging process of SARS-CoV-2 [62]. In this study, we demonstrated that suramin attenuated NiV-induced syncytium formation, suggesting its potential antiviral activity against NiV infection, thus reinforcing the idea of suramin’s broad-spectrum antiviral activity.

### 2.4. In Silico Results Show That Suramin Binds Effectively to NiV-G’s Central Hole and EphrinB2’s G-H Loop

To understand the underlying molecular mechanism and the difference in the fusion inhibitory activity of suramin and OA, molecular docking and MD simulations were conducted. Using the experimentally-resolved NiV-G and human EphrinB2 complex (PDB ID: 2VSM) [63], the two ligands, suramin and OA, were docked to each of the proteins and the residue contact profile of the docking poses was analyzed. The top docking pose of each ligand, indicated by the lowest binding energy, was further simulated in a complex with the NiV-G and the EphrinB2 in the explicit solvent for 10 ns, and the binding free energy was evaluated using MM/GBSA.

**Figure 5:**
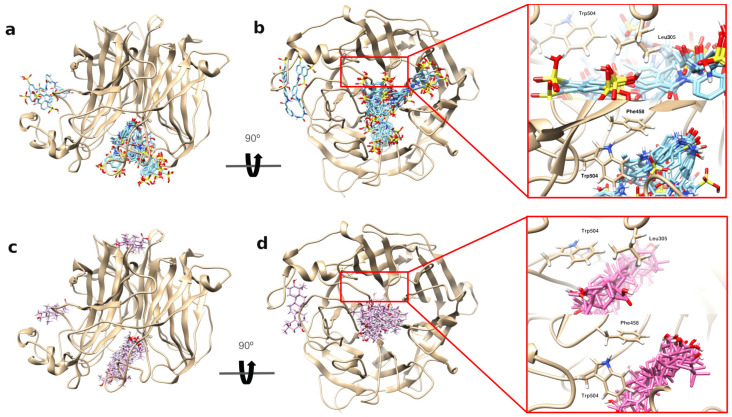
Top 10 docking poses of suramin and OA on NiV-G protein. (**a**,**b**) Top 10 docking poses of suramin on NiV-G. (**c**,**d**) Top 10 docking poses of OA on NiV-G. Most of the top 10 docking poses of suramin and oleanolic acid are clustered inside the central hole of NiV-G. The insets on the rightmost panels highlight key interactions with three critical residues (Leu305, Phe458, and Trp504) in NiV-G’s central hole, which are known to bind EphrinB2. Suramin and OA were shown interacting with these residues, providing further insight into their binding with NiV-G.

The docking results showed that suramin has a higher binding affinity towards NiV-G and EphrinB2 as compared to that of OA. An analysis of both of the ligand’s top 10 docking poses with NiV-G showed that both of the ligands bind in the central hole of the NiV-G (Figure 5). This central hole was previously identified to be the binding site of EphrinB2’s G-H loop (Figure S1B) [63]. In the experimentally-resolved structure of the NiV-G EphrinB2 complex, specific residues within the central hole, such as Leu305, Phe458, and Trp504, were identified to interact with EphrinB2’s G-H loop, particularly at Leu124 and Trp 125 [63]. Among the three NiV-G residues, only Leu305 was identified as one of the top 20 residues contributing to the enthalpic binding of NiV-G to EphrinB2 (Table S1). A closer analysis of these NiV-G residues reveals that suramin’s docking poses closely interact with them, especially with Leu305. Notably, suramin has twice the contact probability with Leu305 compared to OA’s (Figure S7). This suggests that Leu305 may be a crucial residue for both suramin and EphrinB2 binding. Our findings indicate that suramin might interfere with EphrinB2’s binding by interacting with the key residues within NiV-G’s central hole, especially with Leu305.

**Figure 6:**
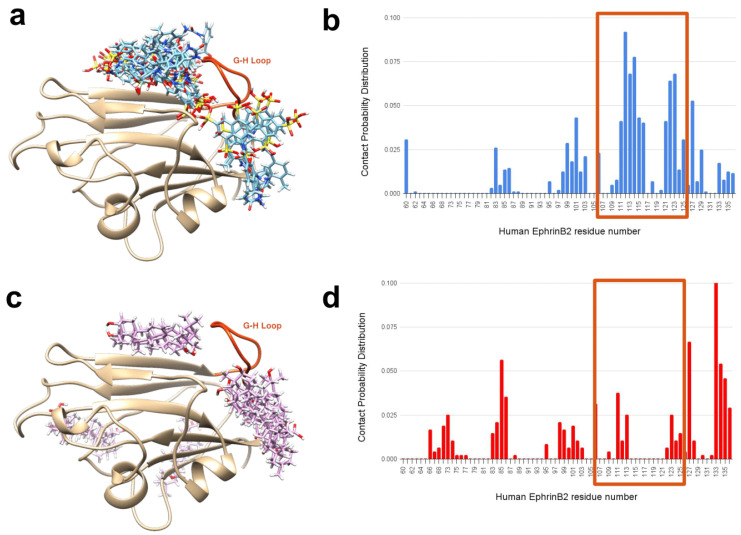
Top 10 docking poses of suramin and OA on human EphrinB2 and its contact distribution profile. (**a**,**c**) The top 10 docking poses of suramin closely cluster around EphrinB2’s G-H loop (residues 107–125, highlighted in red-orange in panel (**a**,**c**), in contrast to OA’s top 10 docking poses (panel **c**). This observation is further substantiated by the contact probability distribution illustrated in panel (**b**), revealing that the top 10 docking poses of suramin primarily engage with the G-H loop. The residues corresponding to the G-H loop are enclosed within an orange rectangle, highlighting a significant interaction. This stands in contrast to the contact probability distribution for OA’s poses depicted in panel (**d**). This contact probability distribution per residue is defined as the ratio of the number of heavy atoms of a specific EphrinB2 residue contacted by the ligand (i.e., suramin and OA) and the total number of heavy atoms contacted by the ligand for all the residues in EphrinB2. Further details on calculating this profile can be found in the Section 3.6.

When examining EphrinB2’s interaction with suramin and OA, it is notable that suramin’s top 10 docking poses clustered mainly around the EphrinB2’s G-H loop (Figure 6a), wherein OA was observed to bind distantly to this loop (Figure 6c). Our contact distribution profiles further reveal that suramin has the highest contact probability with G-H loop residues (enclosed in red-orange box) (Figure 6b). In contrast, OA’s contact distribution profile shows that the ligand rarely interacts with the G-H loop’s residues (Figure 6d). This result shows that another mechanism by which suramin can inhibit EphrinB2’s binding with NiV-G is by binding to the G-H loop, thereby preventing its embedment into NiV-G’s central hole.

To identify the residues that contribute to the enthalpic binding energy of these ligands, the top docking pose with the lowest binding energy for each ligand was simulated in a complex with NiV-G and EphrinB2. The MM/GBSA-calculated free binding energy shows that suramin has a higher binding affinity as compared to OA for both NiV-G and EphrinB2 (Table 1). Intriguingly, 12 of the top 20 residues in EphrinB2 that contribute to the enthalpic binding of suramin were also the same residues that contributed to the enthalpic binding of NiV-G (Table S2). In conclusion, the findings suggest that suramin demonstrated fusion inhibitory activity against NiV in vitro, possibly by binding to NiV-G’s central hole or EphrinB2’s G-H loop, blocking the NiV-G and EphrinB2 complexation.

## 3. Materials and Methods

### 3.1. Cell Culture and Compound Preparation

The *Spodoptera frugiperda* (Sf21) cell (IPLB-Sf21-AE) was cultured in Hink’s TNM-FH medium (Caisson Laboratories, Inc., Smithfield, RI, USA) containing 8% heat-inactivated fetal bovine serum (FBS) (Gibco, Thermo Fisher Scientific, Madison, WI, USA). The human embryonic kidney (HEK239) cell (ATCC-CRL-1573) was maintained in Dulbecco’s Modified Eagle Medium (Gibco, Thermo Fisher Scientific, USA) supplemented with 10% FBS and incubated at 37 °C supplemented with 5% CO_2_. Phytochemical compounds were purchased from Sigma-Aldrich (St. Louis, MO, USA) (Oleanolic acid-#O5504, Baicalin-#572667, Baicalein-#465119, and Suramin-#S2671).

### 3.2. Preparation of Donor Plasmid and Generation of Recombinant Baculoviruses

A polycistronic baculovirus expression vector for expressing NiV-F (GenBank: **NP_112026**) and NiV-G (GenBank: **NP_112027**) protein was chemically synthesized and codon-optimized for insect cells (MDBio, Taipei City, Taiwan). The NiV-F was cloned into *Eco*RI- and *Nhe*I-digested pFB-polH-MCS-PnV339-EGFP-Rhir-MCS (Figure S8A), generating pFB-polH-NiVF-PnV339-EGFP-Rhir-MCS. The NiV-G was subsequently cloned into *Spe*I- and *Xba*I-digested pFB-polH-NiVF-PnV339-EGFP-Rhir-MCS. The final vector was termed pFastBac-polh-NiVF-PnV339-EGFP-Rhir-NiVG (Figure 1A).

The sequence for EphrinB2 was derived from the HEK239 cell. The total RNA of HEK cells was extracted using the SV Total RNA Isolation System Kit (Promega, Madison, WI, USA) and was converted to cDNA using the GoScript™ Reverse Transcription System Kit (Promega, USA). Both procedures were performed according to the manufacturer’s protocol. The EphrinB2 sequence was amplified from the cDNA template using 5′-AATTGCTAGC**CAATTG(*Mfe*I)**CCACCATGGCTGTGAGAAGGGAC-3′ and 5′-GGGCAATT**GTCGAC(*Sal*I)**TTA*AGCGTAATCTGGAACATCGTATGGGT(HA-Tag)*AGACCTTGTAGTAAATGTTCGCC-3′. The amplified EphrinB2 was sequenced (Mission Biotech, Taipei City, Taiwan) before ligating into *Eco*RI- and *Sal*I-digested pFastBac-polh-LiuIRES-DsRed2 (Figure S8B), resulting in pFastBac-polh-EphrinB2-LiuIRES-DsRed2 (Figure 1C). Both donor plasmids were used to generate recombinant baculovirus through the Bac-to-Bac system (Invitrogen, San Diego, CA, USA), as described by the manufacturer. The CellfectinII reagent (Invitrogen, USA) was used for bacmid transfection into Sf21 cells described by the manufacturer. The culture was incubated for 5–6 days at 27 °C and examined under a fluorescence microscope (Nikon Eclipse TE2000-U, Tokyo, Japan) using either the FITC filter or the rhodamine filter. The generated recombinant baculoviruses were then amplified in Sf21 cells (1 × 10^7^ cell/flask) and incubated for 5–6 days at 27 °C. The generated recombinant baculoviruses were termed Ac-F-EGFP-G and Ac-EphB2, respectively.

### 3.3. Determination of Target Protein Expression in Infected Insect Cells

Sf21 cells were infected with Ac-F-EGFP-G or Ac-EphB2 using multiplication of infection (MOI) equal to 1 for 4 days at 27 °C. Infected Sf21 cells were lysed using Cytobuster™ (Merck, Rahway, NJ, USA) and centrifuged at 17,000× *g* for 10 min. The supernatant was collected as the cytoplasmic protein. The remaining pellets were then lysed using RIPA buffer (10 mM Tris-HCl, 0.5 mM EDTA, 1% (*v*/*v*) TritonX-100, 0.1% (*w*/*v*) sodium deoxycholate, 0.1% (*w*/*v*) SDS, and 150 mM NaCl) and centrifuged at 17,000× *g* for 10 min to collect the membrane protein. The protein concentration was determined using the Pierce™ BCA Protein Assay Kit according to the manufacturer’s protocol (Thermo Fisher Scientific, USA). The expression of NiV-F tagged with a 6×His peptide, NiV-G tagged with a FLAG (DYKDDDDK) peptide, and EphrinB2 recombinant protein tagged with HA (YPYDVPDYA) peptide was detected by Western blot using anti-His antibody (#600-401-382, Rockland, Rocklin, CA, USA), anti-DDDDK antibody (#ab1162, Abcam, Cambridge, MA, USA), and anti-HA antibody (#GTX115044, GeneTex, Irvine, CA, USA), respectively. The GP64 and GAPDH, as the internal controls, were detected using anti-GP64 antibody (#ab91214, Abcam, USA) and anti-GAPDH antibody (#GTX100118, GeneTex, USA), respectively. The anti-rabbit IgG HRP-conjugated antibody (#7074, Cell Signaling Technology, Danvers, MA, USA) or anti-mouse IgG HRP-conjugated antibody (#31430, Invitrogen, USA) was used as the secondary antibody, and Immobilon™ Western Chemiluminescent HRP Substrate (Millipore, San Francisco, CA, USA) was used as the substrate.

An immunofluorescence assay (IFA) was performed by fixing infected Sf21 cells using 4% (*w*/*v*) paraformaldehyde (Sigma-Aldrich, USA) and the detection of target proteins was performed using the same primary antibodies used for the Western blot analysis. Alexa Fluor^®^ 594- or 488-conjugated AffiniPure anti-rabbit IgG (Jackson ImmunoResearch, West Grove, PA, USA) was used as the secondary antibody.

### 3.4. Syncytium Inhibition Assay

Sf21 cells were seeded in a 96-well plate (4 × 10^4^ cells/well) and infected with Ac-F-EGFP-G and Ac-EphB2 with MOI 3 in each. The medium was replaced with pH-adjusted TNM-FH containing 200 µg/mL cholesterol (#C4951, Sigma-Aldrich, USA) and the tested compounds at 2 dpi. The culture was incubated for another 2 days before syncytium formation was observed. The number of syncytia was counted using three different views of the three wells. The data were analyzed using GraphPad Prism version 8.0.2. using one-way ANOVA.

### 3.5. In Silico Analysis of the Binding of Suramin and Oleanolic Acid on NiV-G and EphrinB2

The molecular docking of suramin and oleanolic acid on NiV-G and EphrinB2 was performed by VINA [64]. Their complexes were subjected to MD simulations, the trajectories of which are analyzed by MM/GBSA [65] to evaluate the binding affinity between compounds and protein receptors. Further information is provided in the Supplementary Information.

### 3.6. Contact Probability Distribution as a Tool for Determining Drug Contact Profile

To further examine the protein-drug interaction by determining the specific residues that a pose mostly interacts with, we calculated the drug’s contact probability distribution profile. To create the contact probability distribution for suramin and OA bound to EphrinB2, we utilized the top 10 docking poses, which are ranked based on the binding free energy. First, we obtained the contact count (*C_i_*) for residue *i*, where *i* goes from 1 to *N* and *N* is the total number of residues in the protein, by counting the number of heavy atoms (atoms other than hydrogen) in the EphrinB2’s residue that contact any heavy atom in a drug pose within a 4 Å cutoff. If we define *Distance*_*a*,*d*_ as the shortest contact distance between a heavy atom *a* of residue *i* in EphrinB2 and a heavy atom of a drug of interest, *C_i_* can be expressed as

(1) $$C_{i}=\sum_{a=1}^{n_{i}}H(4\;Å-{Distance}_{a,d})$$

where *n_i_* is the total number of heavy atoms in residue *i*; *H*(*x*) is a Heaviside step function, which is zero when *x* < 0 and 1 when *x* > 0. We then define $C_{i}^{p}$ as the contact count of residue *i* for the docking pose *p*, which goes from 1 to *M*; *M* is the total number of poses (in our case, *M* = 10). In other words, each residue *i* has *M* values of contact count for each of the *M* docking poses. Therefore, the contact probability, *P_i_*, can be obtained as the contact counts of residue *i* divided by the total contact counts for all residues in EphrinB2 such that

(2) $$P_{i}=\frac{\sum_{p=1}^{M}C_{i}^{p}}{\sum_{i=1}^{N}\sum_{p=1}^{M}C_{i}^{p}}$$

## 4. Conclusions

In this study, we developed a drug screening platform using the BEVS to assess the fusion inhibitory properties of various compounds against the NiV. NiV membrane proteins, NiV-F and NiV-G, along with the EphrinB2 receptor, were successfully expressed on the surface of infected insect cells, enabling the observation of NiV-induced syncytium formation under cholesterol-supplemented conditions. Among the tested compounds, suramin demonstrated the highest fusion inhibitory activity, significantly reducing syncytium formation.

An in silico analysis further suggested that suramin can disrupt the interaction between NiV-G and EphrinB2 by binding either inside the NiV-G’s central hole or on EphrinB2’s G-H loop. Both mechanisms could prevent the interaction between NiV-G and EphrinB2, thereby inhibiting the conformational changes in NiV-F to initiate membrane fusion, as evidenced by the reduced number of syncytia following suramin treatment. These findings indicate that suramin could be a potential therapeutic option against NiV infection. Additionally, our results highlight the utility of BEVS as a viable alternative for developing fusion-based drug screening platforms, as previously demonstrated for the Chikungunya and Zika viruses [66,67].

## Figures and Tables

**Table 1:** The MM/GBSA-calculated free binding energy for suramin and oleanolic acid.

	Binding Energy (kcal/mol)	
	Suramin	Oleanolic Acid (OA)
NiV-G	−49.57 ± 1.00	−17.52 ± 0.48
EphrinB2	−32.69 ± 0.69	−27.21 ± 0.66

## Data Availability

The original contributions presented in the study are included in the article and Supplementary Materials, further inquiries can be directed to the corresponding authors.

## Supplementary Materials

The following supporting information can be downloaded at: https://www.mdpi.com/article/10.3390/ijms25169102/s1. References [63,64,68,69,70,71,72,73,74,75,76,77] are mentioned in Supplementary Materials.
