# Supplementary material for: Development of Fusion-Based Assay as a Drug Screening Platform for Nipah Virus Utilizing Baculovirus Expression Vector System
Source: Int J Mol Sci. 2024 Aug 22;25(16):9102. doi: 10.3390/ijms25169102 (PMC11354753; doi:10.3390/ijms25169102)
Supplement: Supplementary file 1 [file ijms-25-09102-s001.zip › ijms-3070815-supplementary.pdf]

SUPPLEMENTARY FIGURE

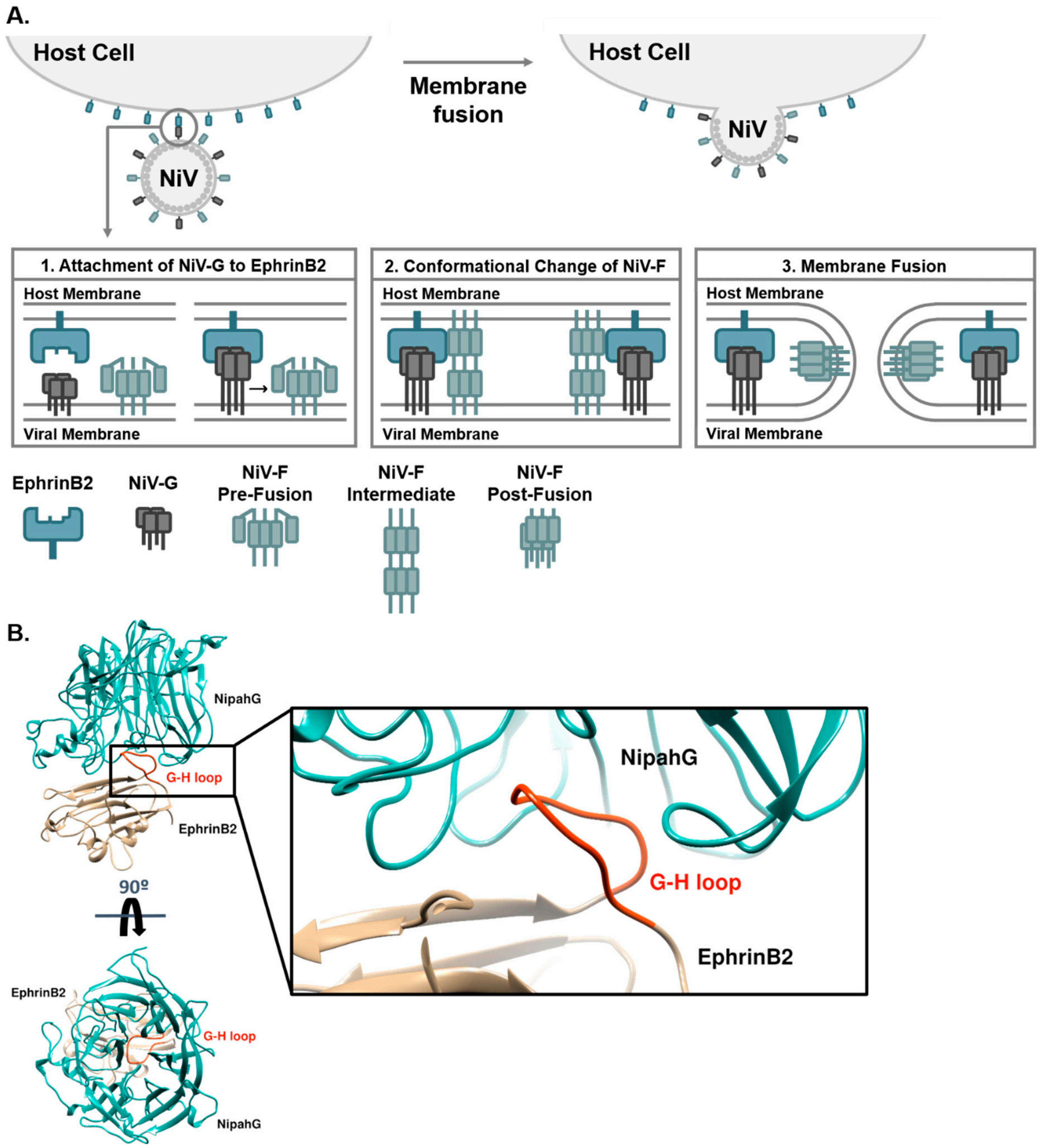

Figure S1. (A) Schematic illustration of NiV-induced membrane fusion. (B) 3D representation of the NiVG-EphrinB2 complex. This depicts the complexation between the NiVG protein and human EphrinB2. EphrinB2's G-H loop interacts with the residues at the central hole of the NiV-G protein.

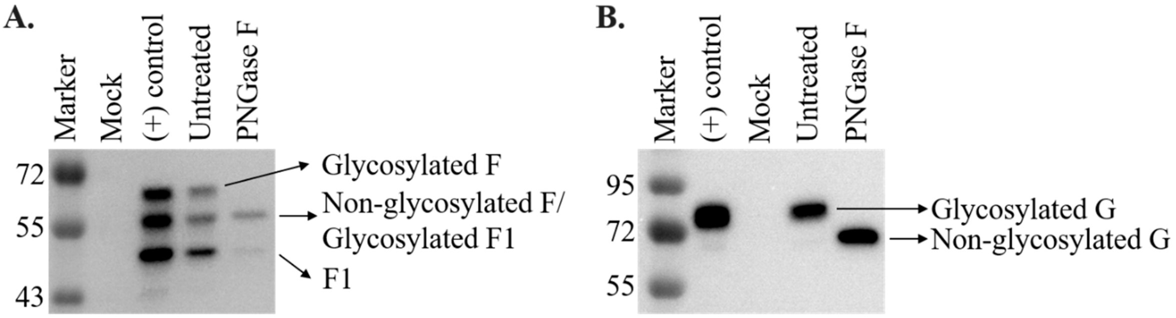

Figure S2. Removal of glycosylation from recombinant NiV-F and NiV-G expressed in infected Sf21 cells. Sf21 cells were infected by Ac-F-EGFP-G with MOI 1. Total cytosolic protein was collected at 4 dpi. The glycosylation was removed using PNGase-F enzyme (New England Biolabs, Massachusetts, USA) according to the manufacturer's protocol. Western blot was carried out to analyze the result. (A) Western blot result against anti-his antibody (NiV-F). (B) Western blot result against anti-DDDDK antibody (NiV-G).

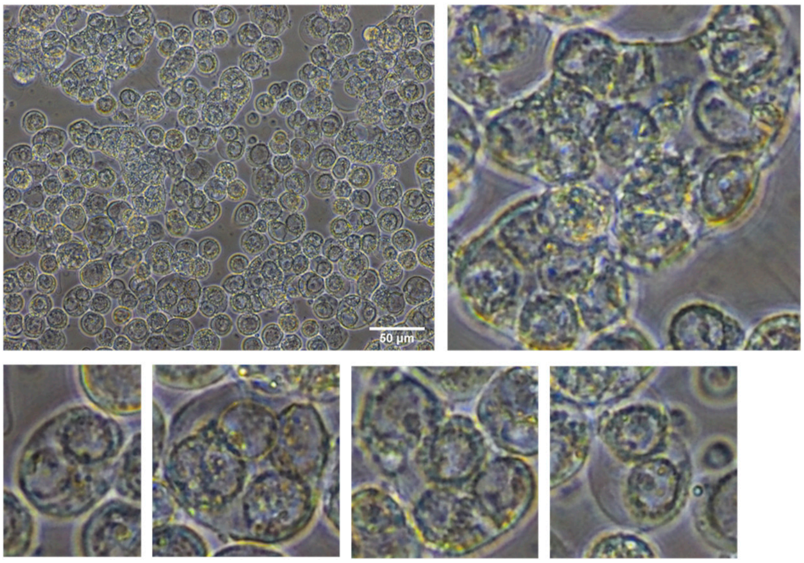

Figure S3. Example of syncytium formation. All multinucleated cells were counted as 1 syncytium formation. Quantification analysis was done by counting all the syncytium formations observed in 5 different views in the same well. Scale bar for the top left figure is equal to 50 μm.

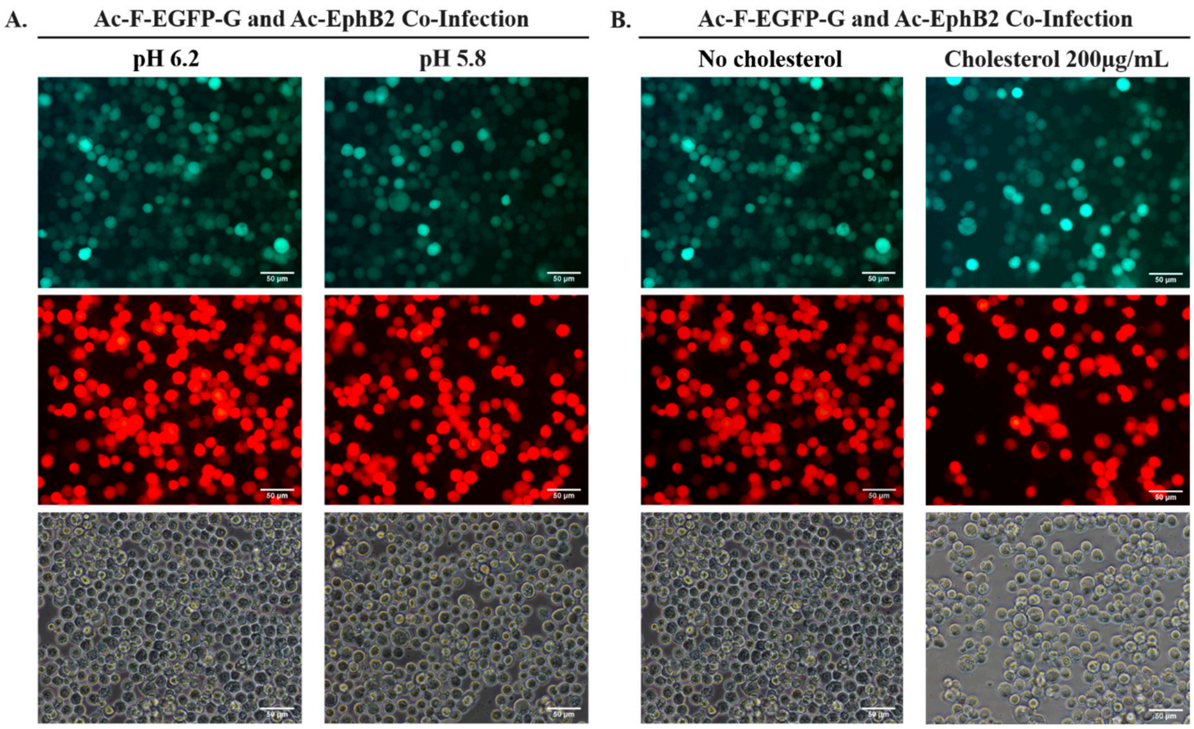

Figure S4. Observation of NiV-induced syncytium in Ac-F-EGFP-G- and Ac-EphB2-co-infected Sf21 cells. (A) Comparison of NiV-induced syncytium in Ac-F-EGFP-G- and Ac-EphB2-co-infected Sf21 cells cultured in TNM-FH medium adjusted to pH 6.2 and pH 5.8 showed no syncytium formation at either pH level. (B) Comparison of NiV-induced syncytium in Ac-F-EGFP-G- and Ac-EphB2-co-infected Sf21 cells cultured in TNM-FH medium with no cholesterol supplementation and with 200 μg/mL of cholesterol supplementation showed no syncytium formation under either condition. Scale bar = 50 μm.

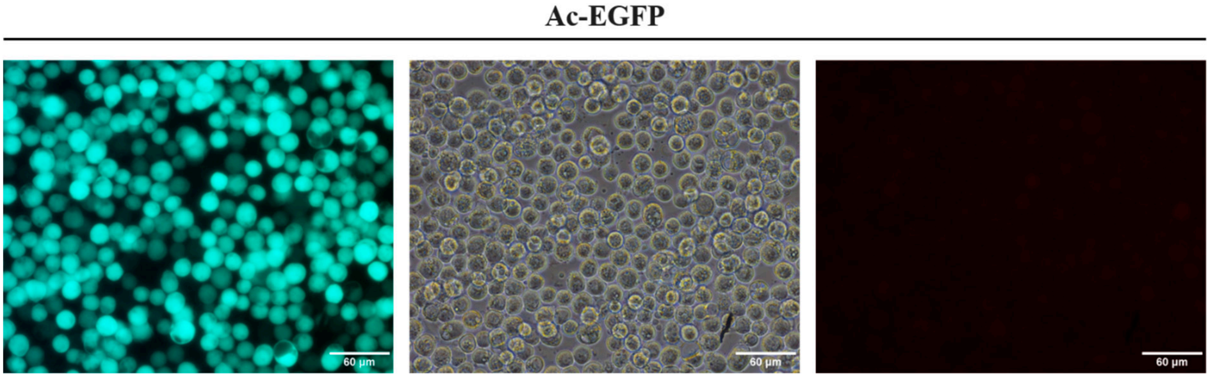

Figure S5. Ac-EGFP-infected Sf21 cells cultured in TNM-FH at pH 5.8 with 200 μg/mL of cholesterol supplementation showed no GP64-induced syncytium formation. Scale bar = 60 μm.

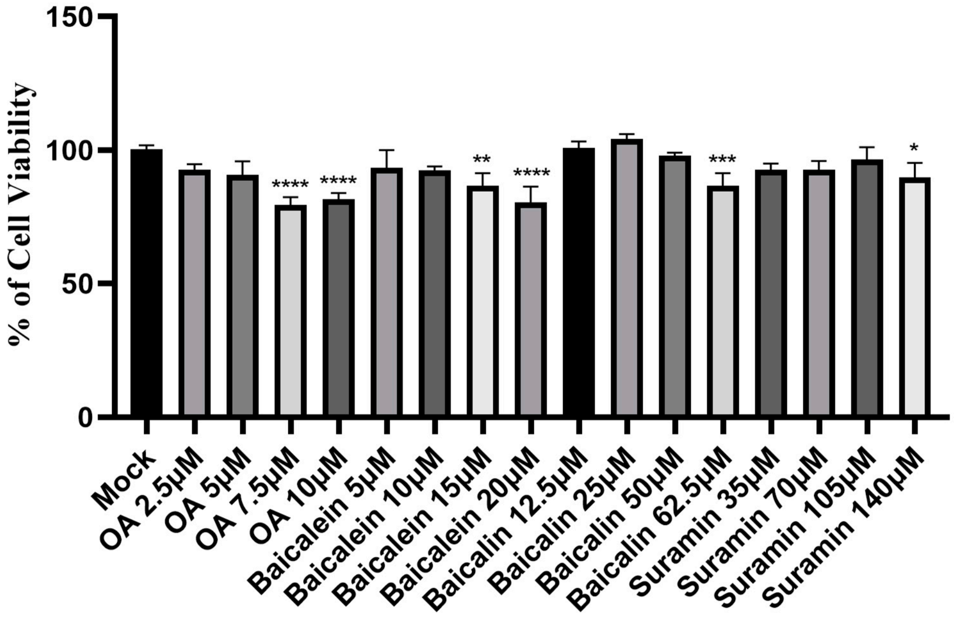

Figure S6. Cytotoxicity assay. Sf21 cells were seeded into 96-well plates at a density of 4 × 10^4 cells per well and cultured in TNM-FH medium adjusted to pH 5.8, supplemented with 200 µg/mL of cholesterol. The cells were then treated with varying concentrations of oleanolic acid (OA), baicalein, baicalin, and suramin, with each treatment performed in triplicate. After a 2-day incubation period, the medium was replaced with 100 µL of 1 mg/mL MTT (3-(4,5-dimethylthiazol-2-yl)-2,5-diphenyltetrazolium bromide) reagent (Invitrogen™), mixed with 100 µL of TNM-FH medium. The plates were protected from light and incubated at 27°C for 2 hours. Following incubation, the MTT solution was removed, and 100 µL of DMSO was added to each well to solubilize the formazan crystals. The plates were then placed on an orbital shaker for 15 minutes in the dark. Absorbance was measured at 570 nm using a microplate reader, and cell viability was calculated based on the absorbance values. * $p < 0.05$, ** $p < 0.01$, *** $p < 0.001$, **** $p < 0.0001$.

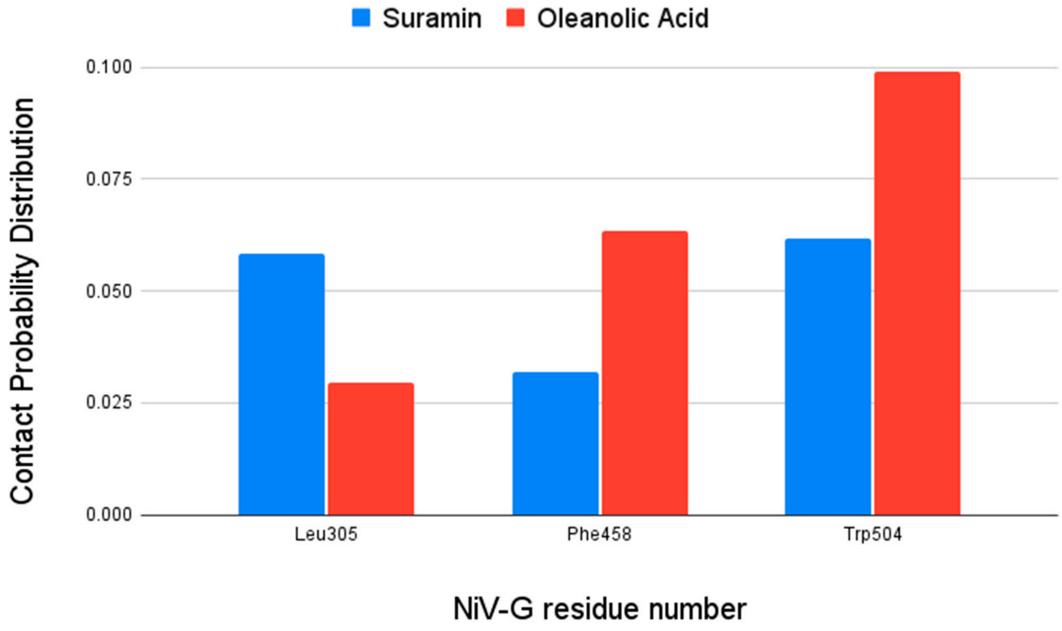

Figure S7. Suramin's and Oleanolic Acid's contact distribution profile with key residues in NiV-G's central hole, such as Leu305, Phe458, and Trp504 that bind to EphrinB2's G-H loop. Details on calculating this profile can be found in the Methodology Section 3.6.

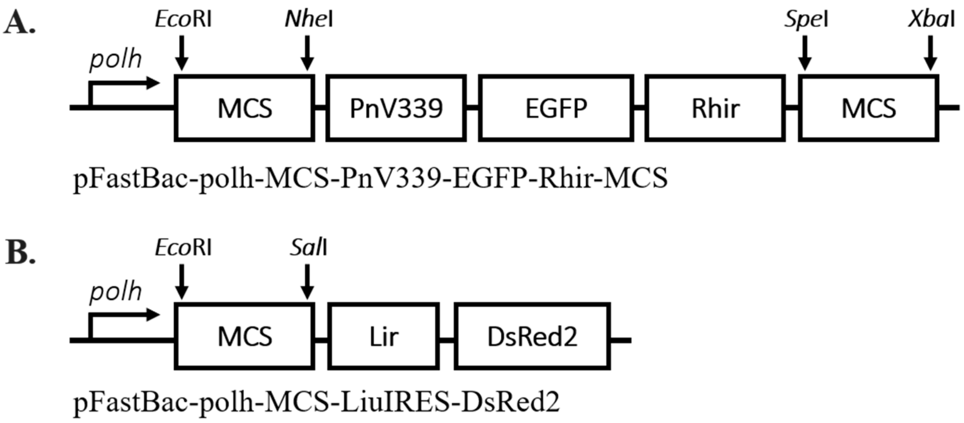

Figure S8. (A) Schematic map of pFB-polh-MCS-PnV339-EGFP-RhiR-MCS. (B) Schematic map of pFB-polh-MCS-Lir-DsRed2.

Method for the *In silico* Analysis

1. Molecular docking

Autodock Vina [1] was used to do the drug screening for NiV-G:EphrinB2 structures (PDB ID: 2VSM) [2]. The docking box was set such that the whole protein was covered. The number of poses and exhaustiveness were set to 20 and 50, respectively.

2. System Setup

Before docking and MD simulations, the protonation state and net charge of the proteins and the ligands were calculated using PDB2PQR [3] at pH 5.8. Moreover, the ligands were parameterized using Antechamber [4]. For MD simulations, each initial structure (NiV-G:EphrinB2 complex or NiV-G/EphrinB2:ligand complex) was prepared using ff14SB [5] forcefield for proteins and solvated with TIP3P water model [6] through tleap [7] from AmberTools20 [8]. Counter ions were added to neutralize the system. Additional ions were further added to reach the 0.15M NaCl concentration.

3. Energy minimization, equilibration, and MD simulations of the NiV-G:EphrinB2 complex

The protein-protein complex system was first subjected to a two-stage energy minimization. For the first stage, harmonic positional restraint of 100 kcal/mol A^2 was applied to all the protein's heavy atoms. For the second stage, only the protein's Cα atoms were applied with harmonic positional restraints of 2 kcal/mol A^2.

The energy-minimized system was heated from 50K to 320K and cooled down from 320K to 310K for 1ns in an isothermal-isochoric (NVT) ensemble. The system was further equilibrated in an isothermal-isochoric (NVT) ensemble for 1ns at 310K. This was followed by an isothermal-isobaric (NPT) ensemble for 5ns at 310K and 1 atm. The constant pressure and temperature were maintained using Berendsen barostat [9] and Langevin thermostat [10], respectively. After equilibration, the system was subjected to a 300ns production run at 2 fs time step applying the SHAKE constraint algorithm [11] to hydrogen atoms in an

isothermal–isobaric ensemble at 310 K and 1 atm. Long-range electrostatic forces were calculated using the Particle Mesh Ewald method [11] with a cutoff distance of 10Å. Before releasing the harmonic restraints on the protein's Cα atoms during the production run, harmonic restraints of 2 kcal/mol A^2 on the protein's Cα atoms were applied during the NVT heating and equilibration, and NPT equilibration stages.

4. Energy minimization, equilibration, and MD simulations of the NiV-G/EphrinB2 in complex with a ligand

For the protein-ligand complex simulation, the pose of the ligands with the lowest binding energy in the docking results was chosen as the initial ligand structure. The prepared complex system was subjected first to energy minimization with two stages. For the first stage, harmonic positional restraints of 50 kcal/mol A^2 were applied to all the protein's and ligand's heavy atoms. For the second stage, the protein's Cα atoms and the drug's heavy atoms were applied with harmonic positional restraints of 10 and 2 kcal/mol A^2, respectively.

The energy-minimized system was heated from 50K to 320K and cooled down from 320K to 310K for 1ns in an isothermal-isochoric (NVT) ensemble while applying harmonic restraints of 2 and 1 kcal/mol A^2 on protein's Cα atoms and drug's heavy atoms, respectively. The system was further equilibrated in an isothermal-isochoric (NVT) ensemble for 1ns at 310K while maintaining the applied harmonic restraints on the specified atoms. This was followed by an isothermal-isobaric (NPT) ensemble for 2ns at 310K and 1 atm while relaxing the applied harmonic restraints to 1 and 0.1 kcal/mol A^2 on the protein's Cα atoms and the drug's heavy atoms, respectively. The constant pressure and temperature were maintained using Berendsen barostat [12] and Langevin thermostat [9], respectively. After equilibrating the system, the system was subjected to 10 ns production run at 2 fs time step applying the SHAKE constraint algorithm [10] to hydrogen atoms in an isothermal–isobaric ensemble at 310 K and 1 atm. Long-range electrostatic forces were calculated using the Particle Mesh Ewald method [11] with a cutoff distance of 10Å.

Table S1. Top 20 NiV-G residues that contribute to the enthalpic binding towards EphrinB2.

Residue name + ID*
ARG 242
ALA 532
GLU 533
THR 531
CYS 240
ASN 557
TYR 581
GLN 530
SER 239
SER 241
GLN 559
THR 556
GLU 505
ASP 555
GLU 554
LEU 305
ARG 402
THR 583
ILE 588
VAL 507

* These residues are listed in descending order based on their enthalpic binding energy contribution towards EphrinB2. Among these top 20 residues, only Leu305 was found in the experimentally-resolved structure (PDB ID: 2VSM) to be a key residue in the NiV-G binding with EphrinB2.

Table S2. Top 20 EphrinB2 residues that contribute to the enthalpic binding of NiV-G, suramin, and oleanolic acid (OA).

NipahG	Suramin	OA
LYS 116	THR 114	MET 83
PHE 120	GLN 118	TYR 135
LYS 63	SER 121	ASP 134
GLN 118	PHE 113	LYS 133
PRO 122	LYS 116	VAL 84
PHE 113	PRO 122	LYS 86
THR 114	ILE 115	GLY 126
LYS 131	PHE 120	ASP 85
LEU 124	ASN 123	TRP 125
ILE 60	PHE 129	TYR 136
ILE 34	THR 99	ASN 98
GLY 61	LYS 112	GLN 130
GLU 128	LEU 101	LEU 127
TRP 125	MET 83	PHE 129
ASN 123	LEU 124	LEU 124
SER 121	PHE 117	TYR 82
GLU 119	LEU 127	VAL 167
ILE 111	LYS 60	ILE 137
VAL 35	LEU 61	LEU 101
LYS 112	LEU 102	LEU 164

* Among the top 20 EphrinB2 residues that contribute to the binding of NiV-G, 12 of them (highlighted in grey) are the same residues that bind suramin as well. Meanwhile, only 3 residues (highlighted in grey) are present for oleanolic acid.

References

1. Trott, O. and A.J. Olson, *AutoDock Vina: improving the speed and accuracy of docking with a new scoring function, efficient optimization, and multithreading.* Journal of computational chemistry, 2010. **31**(2): p. 455-461.
2. Bowden, T.A., et al., *Structural basis of Nipah and Hendra virus attachment to their cell-surface receptor ephrin-B2.* Nature structural & molecular biology, 2008. **15**(6): p. 567-572.
3. Dolinsky, T.J., et al., *PDB2PQR: expanding and upgrading automated preparation of biomolecular structures for molecular simulations.* Nucleic acids research, 2007. **35**(suppl_2): p. W522-W525.
4. Wang, J., et al., *Automatic atom type and bond type perception in molecular mechanical calculations.* Journal of molecular graphics and modelling, 2006. **25**(2): p. 247-260.
5. Maier, J.A., et al., *ff14SB: improving the accuracy of protein side chain and backbone parameters from ff99SB.* Journal of chemical theory and computation, 2015. **11**(8): p. 3696-3713.
6. Joung, I.S. and T.E. Cheatham III, *Molecular dynamics simulations of the dynamic and energetic properties of alkali and halide ions using water-model-specific ion parameters.* The Journal of Physical Chemistry B, 2009. **113**(40): p. 13279-13290.
7. Daoudi, S., et al., *Electronic structure and optical properties of isolated and TiO2-grafted free base porphyrins for water oxidation: A challenging test case for DFT and TD-DFT.* Journal of Computational Chemistry, 2019. **40**(29): p. 2530-2538.
8. Case, D.A., et al., *Amber 2021.* 2021: University of California, San Francisco.
9. Pastor, R.W., B.R. Brooks, and A. Szabo, *An analysis of the accuracy of Langevin and molecular dynamics algorithms.* Molecular Physics, 1988. **65**(6): p. 1409-1419.
10. Hopkins, C.W., et al., *Long-time-step molecular dynamics through hydrogen mass repartitioning.* Journal of chemical theory and computation, 2015. **11**(4): p. 1864-1874.
11. Darden, T., D. York, and L. Pedersen, *Particle mesh Ewald: An N · log (N) method for Ewald sums in large systems.* The Journal of chemical physics, 1993. **98**(12): p. 10089-10092.
12. Berendsen, H.J., et al., *Molecular dynamics with coupling to an external bath.* The Journal of chemical physics, 1984. **81**(8): p. 3684-3690.
